# Primary cardiac lymphoma with isolated parenchymal central nervous system relapse: report of two cases and review of the literature

**DOI:** 10.3332/ecancer.2014.474

**Published:** 2014-10-23

**Authors:** Juan Montoro, Lucia Mattia, Paola Bertazzoni, Sarah Liptrott, Nicola Colombo, Maurizio Civelli, Lorenzo Preda, Daniele Laszlo, Giovanni Martinelli, Niccolò Frungillo

**Affiliations:** 1 Division of Clinical Haematology/Oncology, European Institute of Oncology, Via Ripamonti 435, Milan 20141, Italy; 2 Division of Cardiology, European Institute of Oncology, Via Ripamonti 435, Milan 20141, Italy; 3 Division of Radiology, European Institute of Oncology, Via Ripamonti 435, Milan 20141, Italy

**Keywords:** primary cardiac lymphoma, central nervous system, diffuse large B-cell lymphoma

## Abstract

Primary cardiac lymphoma (PCL) is a rare subset of non-Hodgkin’s lymphoma involving the heart and/or pericardium with no or minimal evidence of extracardiac involvement at presentation. Distant relapses have infrequently been observed.

We report two cases of this disorder that showed isolated central nervous system (CNS) relapse. Diagnosis by endomyocardial biopsy was consistent with diffuse large B-cell lymphoma. After immunochemotherapy they achieved complete remission (CR). Eight and five weeks after, isolated CNS relapses were observed respectively. The first patient was treated with high-dose methotrexate (HD-MTX) and high-dose cytarabine, resulting in a second CR. She then went onto receive autologous stem-cell transplantation but unfortunately died shortly after because of infection. The second patient received systemic CNS prophylaxis with HD-MTX, and later she was treated with an induction chemotherapy strategy with evidencing of progressive disease after two courses of treatment. She was subsequently initiated on a salvage therapy with cytarabine, followed by whole-brain radiotherapy, and autologous stem-cell transplant (ASCT), finally achieving a complete remission.

Isolated CNS relapse is a very uncommon pattern in PCL and a standard approach to treatment is not yet well established. Nevertheless, the importance of CNS evaluation, using magnetic resonance imaging (MRI) and lumbar puncture, in patients with PCL should be considered, and further studies are recommended to determine the appropriate management of this complication.

## Case 1

A 54-year-old woman presented to another hospital in January 2011 with a one-month history of dyspnoea. Her electrocardiogram (ECG) was in sinus rhythm with low-voltage QRS intervals. Six months later, the patient presented with worsening heart failure symptoms. Evaluation by echocardiogram and cardiac magnetic resonance imaging (MRI) showed a reduction of systolic left ventricular function. Laboratory findings revealed elevated markers for myocardial injury and hypereosinophilia. To exclude Loeffler endocarditis, the patient underwent an endomyocardial biopsy. However, the histology revealed a myocardial localisation of diffuse large B-cell lymphoma (DLBCL). Staging by full-body computed tomography (CT) scan, positron emission tomography (PET)/CT, and bone marrow biopsy did not show extracardiac involvement of lymphoma. Common laboratory and serologic tests were unremarkable, and in particular the patient was human immunodeficiency virus (HIV) negative.

The patient was diagnosed with a primary cardiac lymphoma (PCL) and received chemotherapy with etoposide, doxorubicin, cyclophosphamide, vincristine, prednisone, and bleomycin (VACOP-B) in combination with rituximab for 12 weeks. No prophylaxis for central nervous system (CNS) dissemination was performed. After treatment, she achieved a complete remission (CR).

Two months after completion of chemotherapy, the patient presented with a three-day history of headache and vomiting. A MRI examination of the brain, revealed an infiltrating lesion slightly hyperintense on T2 weighted images adjacent to the fourth ventricle (Figure 1A). Cerebrospinal fluid (CSF) analysis revealed the absence of lymphomatous leptomeningeal involvement. A systemic evaluation was conducted without evidence of other location of disease, and she was transferred to our hospital.

She received chemotherapy with high-dose methotrexate (HD-MTX) in combination with high-dose cytarabine (HD-Ara-C): MTX dose of 3 g/m^2^ every two weeks for two cycles plus six doses of Ara-C at 2 g/m^2^ every 12 hours. The patient achieved second CR (Figure 1B) and was consolidated with autologous stem-cell transplant (ASCT). Unfortunately, she died because of a pulmonary infection in the pre-engraftment phase.

## Case 2

**Figure 2.**
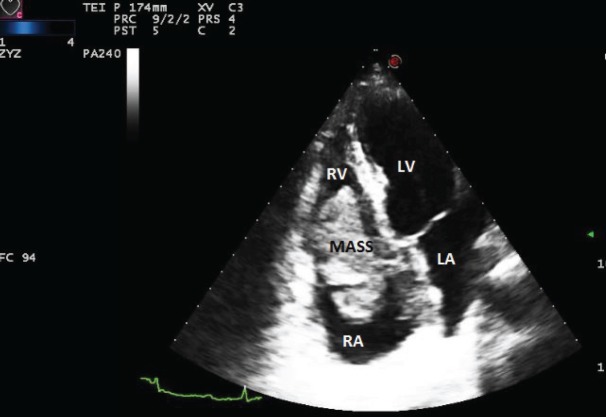
The transthoracic echocardiogram showed a mass located between the right atrium and the right ventricle. LV: left ventricle, LA: left atrium, RV: right ventricle, and RA: right atrium.

A 59-year-old woman presented to another hospital in April 2013 because of one month history of dyspnoea and chest pain. A transthoracic echocardiogram showed a mass twisting between the right atrium and ventricle (Figure 2). With the suspicion of a malignant cardiac tumour, a cardiac catheter-guided endomyocardial biopsy was performed; the pathologic findings and immunophenotypic studies revealed a DLBCL. A complete blood count and serology was unremarkable; and also this patient was immunocompetent. A full-body CT was negative for distant extracardiac sites of lymphoma, and the bone marrow biopsy was unremarkable. PET/CT was not performed.

The diagnosis was a PCL, and she was transferred to our institution in a critical condition. The patient was treated with a CHOP-like regimen: R-ACOD (Rituximab, Adriamycin, cyclophosphamide, vincristine, and prednisone) every 21 days for eight cycles. Based on our previous experience, we performed a lumbar puncture and a CT scan of the brain which were normal. Nevertheless, we decided to administer CNS prophylaxis with systemic HD-MTX at a dose of 1.5 g/m^2^ administered every two weeks for four cycles.

After the first course of treatment, the patient noted an improvement in her cardiac symptoms. A transthoracic echocardiogram, cardiac MRI, CT scan, and PET/CT demonstrated complete disappearance of disease after four cycles of chemotherapy.

**Figure 3.**
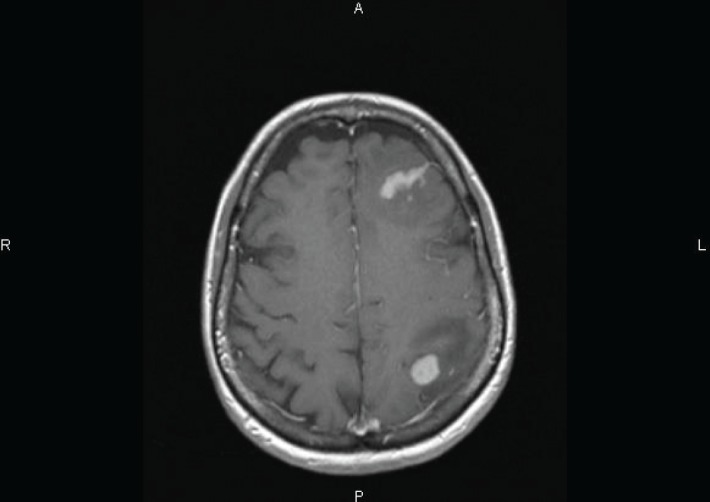
Axial T1-weighted MRI image performed after Gadolinium injection showing two enhancing lesions surrounded by oedema, located in the left frontal and frontoparietal regions.

One week later, the patient presented with headache and motor symptoms. The brain MRI showed two enhancing lesions surrounded by oedema, respectively located in the left frontal and frontoparietal regions (Figure 3). A lumbar puncture with flow cytometry of CSF was negative and systemic evaluation did not reveal any other location of lymphoma.

She received induction chemotherapy with HD-MTX (3 g/m^2^ every two weeks for two cycles) for CNS relapse of lymphoma. However, the follow-up brain MRI after two courses of HD-MTX revealed progressive disease. Therefore, HD-MTX in combination with cytarabine was initiated as salvage therapy without improvement of CNS disease. Whole-brain radiotherapy was than performed with a CR. This patient was then consolidated with ASCT.

## Discussion

We described two cases of PCL which were subsequently followed by extranodal relapse: parenchymal CNS. To date, few cases of PCL with CNS relapse have been described in the literature, and to the best of our knowledge, ours are the first two cases of PCL with isolated CNS recurrence reported by a single center.

PCL is defined as an uncommon extranodal lymphoma involving only the heart and/or the pericardium. A less restrictive definition includes small secondary lesions elsewhere, with the vast bulk of the tumour arising in the heart, and the cardiac involvement by disseminated non-Hodgkin’s lymphoma (NHL) should be excluded [1]. Thus, diagnosis requires clinical and imaging investigations to rule out an alternative site of origin.

PCL should be treated like other aggressive lymphomas arising in other primary sites. In most cases of PCL, lymphoma cells originate from the B-lymphocyte lineage, being DLBCL the most common subtype of PCL [1]. Anthracycline and cyclophosphamide are key therapies for DLBCL [2]. To date, it seems clear that the introduction of a rituximab containing regimen has led to improvement in the overall survival (OS) of patients with PCL [3]; however the optimum schedule of chemotherapy in PCL has not been determined [4].

After the addition of rituximab to standard chemotherapy in patients with systemic DLBCL, the incidence of CNS relapse appears to have decreased [5].

In a retrospective study of 197 cases of PCL, CR was achieved in 59% of patients who were treated by any modality, and 12 patients showed relapse before one year. According to this largest review of PCL to date, there was no case showing CNS recurrence [6]; however in the literature, there are three case reports and all of them, like our two cases, show a constant early recurrence to the CNS (Table 1); all of them were treated with high-dose chemotherapy regimen based on MTX and Ara-C followed by whole-brain radiotherapy.

Usually, CNS relapse is rare in DLBCL; the strongest evidence for high-risk of CNS relapse according to anatomical sites of involvement is for testicular, breast, and involvement of the epidural space. Recent guidelines recommend the use of CNS prophylaxis with directed therapy (intrathecal or systemic) for patients with high-grade NHL presenting risk factors [10]. Many studies have suggested that CNS relapse arises soon after diagnosis and a significant proportion present during therapy or shortly after completion of treatment [11, 12]. Our cases, in agreement with this evidence, suggest that initial CNS subclinical involvement may have gone undetected and also supports the consensus that any planned prophylactic measures should be adopted early during the treatment course. Isolated CNS relapse during the initial six months after treatment for the PCL may reflect the persistence of latent CNS disease rather than dissemination of resistant disease. Unfortunately, we did not perform baseline MRI brain evaluation at diagnosis.

Given the fact that the first patient had a CNS relapse, we believe that the administration of prophylactic treatment to reduce the risk of CNS relapse was then considered as a logical management strategy for the second patient.

The Southwest Oncology Group (SWOG), in an attempt to decrease the CNS relapse rate in diffuse aggressive lymphomas, conducted a prospective trial whereby patients were randomly assigned to receive one of four treatments for CNS prophylaxis. The early occurrence of CNS events suggests that these patients had subclinical disease at initial diagnosis. As such, strategies to better detect and treat patients with subclinical CNS disease at diagnosis was anticipated to result in a decrease in the incidence of CNS relapse [12] but no difference was found in CNS relapse. It was then recommended to administer doses of MTX greater than 3 g/m^2^ if systemic chemotherapy is to be used as CNS prophylaxis [10]. Nevertheless, a retrospective study comparing high-dose versus low-dose of MTX showed a higher risk of CNS relapse (5% versus 4%, respectively) [13]. Moreover, a recent study including 3258 patients with DLBCL with higher clinical risks, treated with standard chemotherapy who achieved CR, were analysed to assess the efficacy of CNS prophylaxis. Approximately one-third of patients received different schedules for CNS prophylaxis, while the remaining patients did not receive CNS prophylaxis. The authors concluded that the use of CNS prophylaxis did not prevent CNS relapse and that the use of prophylaxis did not affect the OS in patients with high clinical risks and poor prognostic factors [14]. These, as well as our second cases, suggest that HD-MTX alone might not be sufficient to protect from CNS relapse.

We suggest that in patients with PCL, an individual patient’s circumstances may dictate an alternative approach because CNS relapse is likely to occur even if there is not a failure of control of systemic disease. Thus, a routine evaluation of CNS should be performed by brain magnetic resonance imaging and lumbar puncture.

In conclusion, studies are recommended to determine the real risk of CNS involvement in PCL, refining the predictors of risk of CNS involvement, and identifying those patients who may benefit from CNS-directed therapy according to more precisely tailored therapeutic protocols.

## Figures and Tables

**Figure 1 A.**
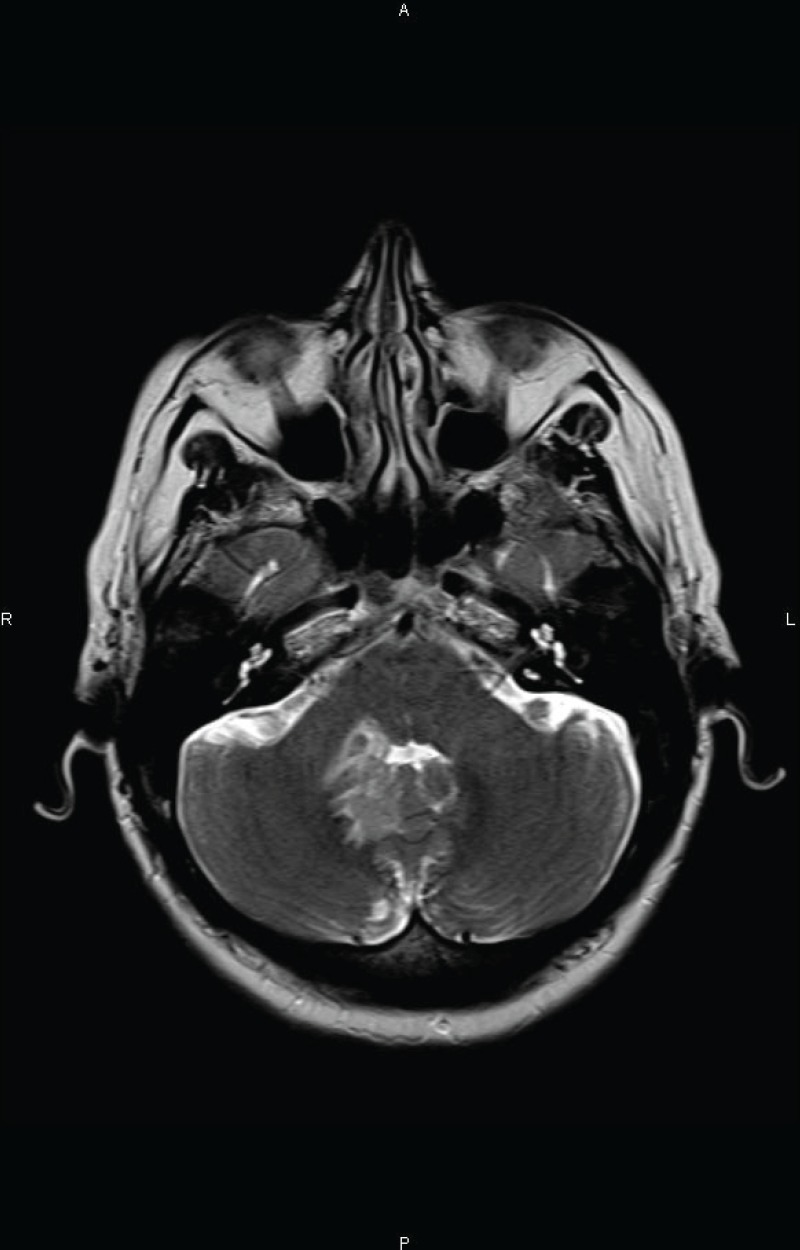
Axial T2 weighted MR image at the level of the posterior fossa revealing a slightly hyperintense infiltrating lesion adjacent to the right side of the fourth ventricle.

**Figure 1 B.**
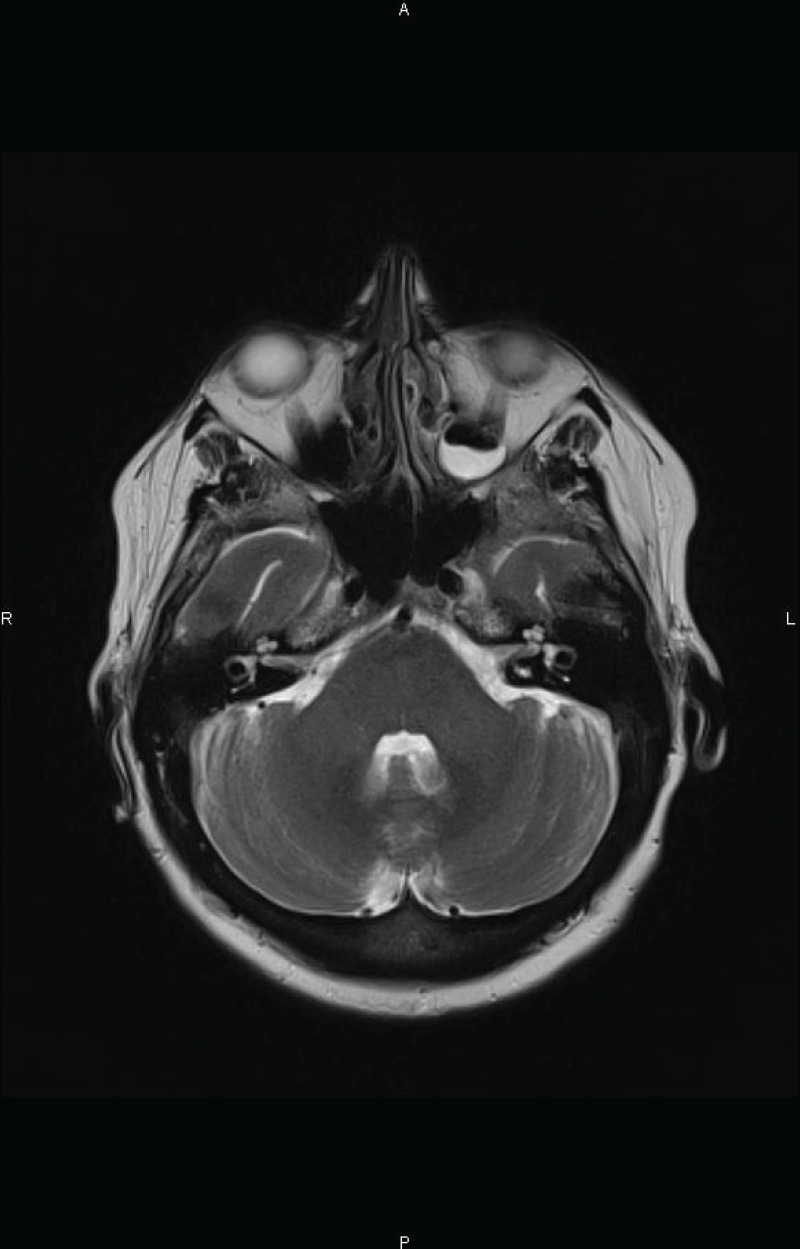
Axial T2 weighted MR image after the conclusion of salvage chemotherapy demonstrating the complete disappearance of the lesion.

**Table 1.** Time to CNS relapse in literature and from our experience.

Authors	Date of editing	Time to CNS relapse
**Montalbetti *et al*** [7]	1999	< one month
**Bulum *et al*** [8]	2007	two months
**Jung *et al*** [9]	2014	one month
**European Institute of Oncology**	1st case	two months
**European Institute of Oncology**	2nd case	one month
